# Intersectional configuration of infant mortality due to malnutrition in Colombia: a mini-review

**DOI:** 10.3389/fpubh.2024.1435694

**Published:** 2024-09-03

**Authors:** Paula A. Taborda Restrepo

**Affiliations:** ^1^Population Health, Fundación Santa Fe de Bogotá, Bogotá, Colombia; ^2^Facultad Nacional de Salud Pública, University of Antioquia, Medellín, Colombia

**Keywords:** intersectionality, infant mortality, gender, child care, malnutrition, ethnic group, poverty

## Abstract

This mini review delves into the complex issue of mortality linked to malnutrition, highlighting its multifaceted nature beyond just biomedical factors, presenting it as an intricate intersectional phenomenon.

**Method:**

The mini-review methodology involved a systematic literature search across databases like PubMed and Scielo, focusing on malnutrition and infant mortality in Colombia. We used specific keywords and Boolean operators to identify relevant studies, emphasizing socio-economic, gender, and ethnic factors, while excluding non-peer-reviewed and outdated publications.

**Results:**

The relationship between gender and food/nutrition has deep historical and cultural roots. Patriarchal norms influence dietary habits based on gender roles, often placing undue responsibility on mothers for children’s nutritional health, reflecting profound social intersections. Mortality due to malnutrition is most prevalent among indigenous and Afro-descendant children in rural, conflict-affected areas with limited access to healthcare. Unpaid domestic work restricts women’s economic independence, intensifying challenges for single-parent households.

**Conclusion:**

A comprehensive understanding can shift institutional attitudes toward mothers, leading to more coherent policy strategies and effective interventions.

## Introduction

Infant mortality due to malnutrition remains a critical issue in many developing countries, and its configuration is influenced by a multitude of socio-economic and contextual variables. This mini review aims to explore the intersectional factors contributing to infant mortality due to malnutrition in Colombia, while also considering comparisons with other Latin American countries. Although the focus is on Colombia, where data is more robust, the patterns observed can offer insights into the broader regional context.

Malnutrition significantly impacts health status and life expectancy in pediatric populations. This review employs an intersectional approach to understand how malnutrition intersects with socio-economic inequalities, particularly gender disparities. The gender variable, which plays a significant role in our discussion, has not been adequately introduced in previous sections. Therefore, this introduction will address this gap, highlighting the importance of considering gender alongside other contextual variables. Moreover, the introduction must captivate readers by clearly identifying the problem and the necessity of conducting intersectional research.

Infant mortality due to malnutrition is an intricate issue that defies simplification. Unlike infectious diseases, hunger spreads not through contagion but through socio-economic disparities. In countries like Colombia, where food is available, deaths from malnutrition underline severe inequities in access to basic resources such as food, water, and healthcare. Despite numerous public policies aimed at addressing these issues, gaps persist due to an economic system that prioritizes private over public interests (1).

The stigma often placed on female caregivers in cases of malnutrition-related deaths is both ethically and politically incorrect. Society assigns women the role of caregivers, yet both parents share responsibility for their children’s wellbeing. The sexual division of labor under modern patriarchy reinforces the notion that caregiving is an innate female function (2). This review aims to explore how malnutrition-related mortality is influenced by deeper structural processes beyond nutritional deficiencies, contributing to the discussion from a critical social theory perspective.

The structure of this article reflects a mini-review format, integrating multiple studies and sources to provide a comprehensive overview of the issue. The mini-review format, as opposed to a full narrative review, allows for a more focused examination of specific themes relevant to infant mortality and malnutrition in Colombia.

## Methods

The methodological approach of this mini-review involves a comprehensive literature search across multiple databases. Databases such as PubMed and Scielo were predominantly utilized to gather relevant studies. PubMed offers access to a vast array of international publications, while Scielo provides critical insights from Latin American research, including studies published in Spanish. In the same way, non-indexed information relevant to the argumentation of the text was included.

Inclusion criteria for this review comprised peer-reviewed articles published between 2000 and 2022 that focus on infant mortality, malnutrition, and socio-economic variables in Colombia and comparable Latin American countries. Exclusion criteria included studies not available in full text and those not relevant to the intersectional analysis of malnutrition and mortality.

Boolean operators were used to refine search queries, ensuring a systematic and replicable approach. Keywords included combinations of Malnutrition, socioeconomic factors, intersectionality, infant mortality, gender.

The search in PubMed yielded 27 papers. Out of these, 26 publications were excluded based on the inclusion criteria defined for this review. From the remaining publications, a final corpus of 1 publication was considered for our review. “Similarly, the search in Scielo returned 7 papers, from which 4 were excluded according to our inclusion criteria, resulting in a final corpus of 3 publications for our review”.

## Results

### Socio-economic inequalities and infant mortality

Socio-economic status is a primary determinant of health outcomes, including infant mortality due to malnutrition. In Colombia, disparities in income, education, and access to healthcare services significantly affect nutritional status and, consequently, infant mortality rates. Studies indicate that lower SES correlates with higher rates of malnutrition and mortality (3, 4).

Colombian rural areas are highly vulnerable to food insecurity and its consequences, such as double burden of malnutrition. Other factors associated with double burden of malnutrition were low mother’s height, mothers with multiple births, households with three or more children under 5 years of age, households with more than seven members, women as the head of the household, indigenous head of the household and child birth spacing of more than 24 months (5). Infant mortality due to malnutrition is a significant concern in Latin America. A study analyzing 20 years of data found Venezuela, Paraguay, and Colombia had the highest percentages of malnutrition-related deaths in children under 1 year. In contrast, Chile had the lowest rates. In Colombia, nearly half of the deaths in children under five are linked to malnutrition, affecting health, education, and economic productivity long-term. Globally, acute malnutrition accounts for 17% of deaths in children under five, with severely malnourished children being 11 times more likely to die than their healthy peers (6, 7).

Between 1998 and 2020, Colombia saw 62,784 deaths in children under five due to malnutrition, averaging eight deaths per day. In 2020, 51% of malnutrition-related deaths were among indigenous or Afro-descendant children, despite these groups representing only 4 and 8% of the population, respectively (8).

Malnutrition is a multifaceted issue that impacts not only the immediate health and survival of children but also their long-term development and the economic growth of their communities. Chronic malnutrition can lead to stunted growth, cognitive impairments, and increased susceptibility to diseases, creating a cycle of poverty and poor health that can span generations. Addressing malnutrition requires a comprehensive approach that includes improving access to nutritious food, healthcare, clean water, and education, as well as addressing underlying social and economic inequalities.

### Food sovereignty and gender

Peasant and popular feminism challenges the capitalist and patriarchal systems by empowering rural, indigenous, and Afro-descendant women to fight for food sovereignty, which encompasses social justice and equality (9). Feminist movements have long advocated for women’s emancipation from unjust realities, emphasizing the need for men and women to freely pursue fulfilling life projects.

Food sovereignty prioritizes local agricultural production, reducing food imports, and supporting internal food marketing chains, which peasant women’s movements have significantly promoted (10). Despite women’s substantial contributions to global agricultural production, they control less land and have limited access to inputs, credit, and services, exacerbating gender disparities in rural development (11).

Women’s participation in unpaid care work limits their economic autonomy, reinforcing gender inequality. In Colombia, women dedicate significantly more time to unpaid care and support than men, affecting their labor market participation and income. This dynamic perpetuates the inferior position of women in the labor market, contributing to a cycle of poverty and dependence (12).

The “feminization of poverty” concept highlights that poverty affects women more acutely than men, with increasing numbers of female-headed households correlating with deteriorating living conditions (13). This trend emphasizes the need to address gendered disparities in access to resources and opportunities. For instance, the burden of unpaid care work falls disproportionately on women, limiting their ability to participate in the formal economy and achieve financial independence. Addressing this requires policies that support women’s economic empowerment, such as providing access to affordable childcare, promoting flexible work arrangements, and ensuring equal pay for equal work (14).

Food sovereignty is positioned as the alternative political approach for rural development in search of guarantees of rights for ways of life in the countryside, especially for the peasantry. With which, it becomes the political platform for the guarantee of human rights in rural areas. Additionally, it seeks to break the dynamics of dependency of the globalizing economic model, questions the model of food and agricultural production, distribution and trade, makes visible the peasant, indigenous and fishing struggle for the right and use of land, seeds, water, as well as, proposes strategies, means and resources for the development and well-being of peasant, rural, fishing, pastoral communities, among others, so that actions are achieved to guarantee decent living conditions necessary for rural communities, and thus, the rights are recognized and needs of the peasantry in this sense, it will seek the organization of communities based on the self-determination of their forms of participation, production and marketing in the food and democratic systems in which they are immersed, respecting their uses, knowledge, and customs for collective and autonomous construction of alternatives for well-being and good living, as well as proposing an innovative vision to build conditions of being and coexisting in and from rurality (15).

Women face greater limitations in accessing agricultural services. The low impact in decision-making spheres, less access to education and poor health make life more difficult. Due to the lack of basic services, their quality of life is so precarious that their domestic tasks are more strenuous than those of poor urban women. These elements of inequality and discrimination constitute forms of violence; and although they have already been recognized in the documents and commitments signed by the Colombian government, they have not been transformed. The mobilization of peasant women to comply with equity policies for rural women did not achieve its goal, although their demands have been supported by official statistics that have shown that, increasingly, rural women increase their labor participation within and outside the agricultural property and constitute strategies to resist poverty (16).

In Colombia, women participate in a much greater proportion in direct care (28.8% compared to 14.4% among men), and the time invested is greater, 2 h and 15 min, compared to 1 h and 28 min for men. Of the total time that the population dedicates to providing unpaid care and support for other members of their households, 76.2% is provided by women and 23.8% by men. In such a way that care and support for people are supported mainly by women. The participation patterns of caregivers in the labor market affect their labor income. Half of caregivers work for pay (51.6%), with a large gender gap: 75.4% among men, and 40.2% among women. Taking care of the home, when carried out only by women, directly benefits men and in turn reinforces the inferior position of women in the labor market (17).

The wage gap between men and women is much greater at lower educational levels. We live in a country with neoliberal policies that, because of dispossession, generate surplus value, in addition to exploitation, oppression and discrimination in its multiple forms (18). In this way, capitalism becomes naturalized and, in doing so, becomes invisible in the eyes of its victims, who even defend private property and the rights to which they are not entitled.

In the 1980s, some feminists began to analyze the phenomenon of poverty from a gender perspective, with the first mention of it in a work by researcher Diana Pearce, from 1978, entitled: The feminization of poverty: Women, work, and welfare. The attention of this work was particularly focused on the description, in statistical terms, that referred to the increase in households headed by women in the United States (which went from 10.1% in 1950 to 14% in 1976, resulting in a 40% increase) and the correlation of this fact with the deterioration of their living conditions, in terms of poverty (by income). There they identified a series of phenomena within poverty that specifically affected women and pointed out that the number of poor women was greater than that of men, that women’s poverty was more acute than that of men and that There was a trend toward a more marked increase in female poverty, particularly related to the increase in female-headed households. To account for this set of phenomena, the concept of “feminization of poverty” was (19).

From another perspective, Chant proposes making visible for analysis what he calls the “feminization of responsibility and obligation,” from a broader context that considers the material conditions of life and the multiple discriminatory processes that must be addressed when considering income gaps, work and living conditions between men and women, which place the latter in situations of poverty. It suggests the need to consider how women increasingly find themselves on the “front lines” and how the burden of family survival falls disproportionately on them. Rescuing the dimension of the “feminization of responsibility” according to the author, aims to convey the idea “that women are assuming greater responsibility in confronting poverty” (20).

Gender disparities play a crucial role in the intersectional configuration of malnutrition and infant mortality. In Colombia, traditional gender roles often place women in less economically stable positions, impacting their ability to provide adequate nutrition for their children. Moreover, female-headed households are disproportionately affected by poverty, increasing the risk of malnutrition and mortality among infants (21).

A study by De Morais Santos et al. (22) on gender inequalities in Brazil provides valuable insights applicable to the Colombian context. The study highlights how socio-cultural constructs of gender roles contribute to economic disparities, affecting women’s access to resources and their children’s health outcomes. This analysis underscores the need for gender-sensitive policies to address malnutrition and reduce infant mortality.

## Intersectionality as an alternative proposal

An intersectional approach to analyzing malnutrition and infant mortality involves examining how various socio-economic, cultural, and gender-related factors intersect to influence health outcomes. In Colombia, this approach reveals that rural–urban disparities, ethnic differences, and access to healthcare services intersect with socio-economic status and gender to shape the nutritional status and mortality rates of infants.

For instance, indigenous and Afro-Colombian communities face higher rates of poverty and limited access to healthcare services, exacerbating the impact of malnutrition on infant mortality. Studies indicate that these communities require tailored interventions that address their specific socio-cultural contexts (21).

A study highlighted the ethnic disparities in malnutrition and infant mortality between indigenous, Afro-Colombian, and mestizo populations. The study found that indigenous and Afro-Colombian infants are disproportionately affected by malnutrition and mortality due to socio-economic marginalization and inadequate healthcare infrastructure in their communities (23). The findings underscore the need for culturally sensitive interventions that address the specific needs of these ethnic groups.

In general, intersectionality refers to the relationship of multiple dimensions of inequalities and forms of oppression on people’s social identity, considering ethnicity, social class, and gender. At the same time, it is understood that the use of triple marginalization perhaps prevents a broader understanding of those other things that affect the situation of indigenous and Afro-descendant women in Latin America (24).

There are certain levels of articulation of social markers in the production and reproduction of social processes of power (domination and oppression) and their impacts on the health-disease process. Intersectionality has emerged as an alternative theoretical-methodological and epistemological approach in analyzes that interrogate the dynamics and complexity of the interactions of social markers at the individual, institutional and structural levels. For intersectionality to be effectively addressed in women’s health studies, both research designs and methodologies must be refined to reflect innovative analytical thinking about gender, equity, and power relations (25). Although historically this concept may come from the declaration of women’s rights, the person who first coined this term in 1989 was the African American lawyer Kimberle Crenshaw (26).

The possibilities generated by intersectionality are part of the new feminist epistemologies that share situated knowledge, relativize the weight of reason in the process of knowledge construction and show how different experiences give rise to different subjectified and intersubjectified plots, which serve to interrogate the paradigm. Positivist that privileges reason and objectivity maintained with the distance between subjects, the emphasis on quantification with measures of central tendency, which for social issues is not the most suitable approach (27).

Just as there is a “second sex,” there is a “second economy.” The work that men have traditionally done is what counts, what defines the global economic landscape. The woman’s work is what comes second, “the other”: all the tasks that he does not perform but on which, at the same time, he depends to be able to carry out his own tasks. To be able to do the work that counts. Adam Smith only half managed to answer the fundamental question of economics. If food was assured, it was not only because the merchants served their own interests through trade. Adam Smith also had it assured because his mother oversaw putting it on the table every day. Nowadays it is sometimes pointed out that the economy is not only founded on an “invisible hand,” but also on an “invisible heart” (2).

## The impact of maternal education on infant nutrition and mortality

Public health policies play a pivotal role in mitigating the effects of malnutrition on infant mortality. Colombia has implemented various programs aimed at improving maternal and child health, yet challenges persist. Evaluations of these programs reveal gaps in implementation and accessibility, particularly among marginalized communities.

Maternal education plays a crucial role in determining infant nutrition and mortality outcomes. Higher levels of maternal education are associated with better nutritional practices, improved healthcare-seeking behavior, and reduced infant mortality rates (28). In Colombia, disparities in educational attainment among women significantly influence the nutritional status of their children.

A study by Viáfara-López et al. (29) examined the impact of maternal education on infant nutrition and mortality in Colombia. The study found that infants born to mothers with higher education levels had lower rates of malnutrition and mortality. The authors attribute this to the increased health literacy, better economic opportunities, and improved access to healthcare services among educated mothers. These findings highlight the importance of promoting female education as a strategy to combat malnutrition and reduce infant mortality.

## Discussion

The findings of this mini-review highlight the multifaceted nature of malnutrition and its impact on infant mortality in Colombia. Socio-economic inequalities, gender disparities, and public health policies are critical factors that intersect to influence health outcomes. Addressing these issues requires comprehensive and intersectional approaches that consider the diverse contexts and needs of affected populations.

The emphasis on gender disparities in this review reflects the significant role that gender plays in shaping socio-economic inequalities and health outcomes. Women’s economic empowerment and gender-sensitive policies are crucial for improving nutritional status and reducing infant mortality. Future research should focus on the specific mechanisms through which gender inequalities affect malnutrition and mortality, incorporating both quantitative and qualitative methods.

Moreover, international comparisons provide valuable lessons for Colombia. The success of community-based nutrition programs and women’s empowerment initiatives in India (28) and other developing countries (22, 27, 30) highlights potential strategies for reducing malnutrition and infant mortality in Colombia. Collaborative efforts and knowledge exchange between countries can enhance the effectiveness of interventions.

Despite extensive regulatory frameworks and significant progress in reducing malnutrition-related mortality, persistent inequalities necessitate alternative approaches. Effective public policies must address the root causes of inequality, empowering women through increased labor participation and reduced wage discrimination. Emphasizing food sovereignty and intersectional analysis in academic and policy discussions can lead to more comprehensive solutions to eradicate unjust and preventable infant mortality (31).

Understanding the multidimensional nature of malnutrition-related mortality can shift institutional perspectives, reducing punitive attitudes toward mothers and fostering more effective intervention strategies. Addressing social inequalities and promoting gender equity are essential to achieving food security and improving health outcomes for all.

In Colombia, it was studied how female empowerment influences child nutrition (32). This should allow for the design of effective public policies for prevention and care for children who are at risk of malnutrition, since there are a series of relevant factors that they potentiate it. It is advisable to review the guidelines of the programs related to the country’s gender equity policy and child nutrition, especially to include within the food and nutritional security plans a component that helps reduce gaps and allows gender equity. Policies should strengthen female autonomy by increasing labor participation and reducing wage discrimination in the labor market.

The implications of thinking about food sovereignty with an intersection of intersections must permeate the academy because in the training and research of mortality associated with malnutrition, there has traditionally been a biological approach that lacks in-depth analysis of this social phenomenon, which is the ultimate result of social inequalities, dispossession, violence, individualism and lack of love, as Cervantes would say: “The greatest opposite that love has is hunger.” This approach from complexity would allow us to propose public policies that aim to eradicate avoidable, unjust, and undignified infant mortality (30).

Inequalities are integrated into the daily life of our country, if policies continue to be proposed that aim to guarantee food security, regardless of whether these do not delve into the roots of food and nutrition problems from a human rights approach. Peoples to have food sovereignty, if we try not to see the pain of those who suffer from gender, class, and race oppression, those roots will never end and this has been the hegemonic approach from the planning of public policies, health care and the prevention methods established in our environment.

## Conclusion

In conclusion, this mini-review underscores the importance of an intersectional approach to understanding and addressing infant mortality due to malnutrition in Colombia. Socio-economic inequalities, gender disparities, and public health policies intersect to influence health outcomes, necessitating comprehensive and context-specific interventions. By integrating international perspectives and focusing on gender-sensitive policies, Colombia can develop more effective strategies to combat malnutrition and reduce infant mortality.

Future studies should explore the specific pathways through which intersectional factors influence health outcomes, employing both quantitative and qualitative methodologies. Additionally, there is a need for continued evaluation of public health programs to ensure their accessibility and effectiveness for marginalized communities. Addressing the root causes of malnutrition and its intersectional dimensions will be crucial for improving health outcomes and reducing infant mortality in Colombia and beyond.
