# Correction: Intersectional configuration of infant mortality due to malnutrition in Colombia: a mini-review

**DOI:** 10.3389/fpubh.2025.1746721

**Published:** 2025-12-09

**Authors:** Paula A. Taborda Restrepo

**Affiliations:** 1 Population Health, Fundación Santa Fe de Bogotá, Bogotá, Colombia; 2 Facultad Nacional de Salud Pública, University of Antioquia, Medellín, Colombia

**Keywords:** intersectionality, infant mortality, gender, child care, malnutrition, ethnic group, poverty

In the published article, there was an error in the **Funding** statement. The funder WWB Foundation Colombia to Paula Andrea Taborda Restrepo was erroneously omitted.

The funding statement appears below:

“The author(s) declare that financial support was received for the research and/or publication of this article. This work was supported by the WWB Foundation Colombia.”

The original version of this article has been updated.

